# The Involvement of TRIB3 and FABP1 and Their Potential Functions in the Dynamic Process of Gastric Cancer

**DOI:** 10.3389/fmolb.2021.790433

**Published:** 2021-12-09

**Authors:** Songyi Liu, Chuxuan Ni, Yizhi Li, Honghao Yin, Chengzhong Xing, Yuan Yuan, Yuehua Gong

**Affiliations:** ^1^ Tumor Etiology and Screening Department of Cancer Institute and General Surgery, The First Hospital of China Medical University, Shenyang, China; ^2^ Key Laboratory of Cancer Etiology and Prevention in Liaoning Education Department, The First Hospital of China Medical University, Shenyang, China; ^3^ Key Laboratory of GI Cancer Etiology and Prevention in Liaoning Province, The First Hospital of China Medical University, Shenyang, China

**Keywords:** TRIB3, FABP1, expression, gastric cancer, biomarker

## Abstract

**Background:** Dysregulated expression of TRIB3 and FABP1 have been previously observed in human cancer tissues. However, there are little information as to their expression change in dynamic gastric diseases and the functional roles.

**Methods:** Tissues from a total of 479 patients, including 89 GS, 102 IM-GA, 144 EGC, and 144 AGC were collected. The protein expressions of TRIB3 and FABP1 were detected by immunohistochemical staining. Meanwhile, the potential functions of TRIB3 and FABP1 in GC were further analyzed by R software and some internet public databases, such as TCGA and DAVID.

**Results:** During this multi-stage process that go through GS to EGC, the expression trend of TRIB3 and FABP1 protein was GS > IM-GA > EGC. Besides, the expression of TRIB3 protein continued to decrease in AGC, while the expression of FABP1 was abnormally increased. *Hp* infection was significantly associated with the decreased expression of TRIB3 and FABP1. In addition, the diagnostic efficiency of the combination of these two indicators to diagnose EGC was higher than that of a single indicator. Survival analysis showed that higher expression of TRIB3 or FABP1 could indicate a better prognosis of GC. The protein expressions of TRIB3 and FABP1 were significantly positively correlated. Moreover, CEACAM5 and PRAP1 were positively correlated with both TRIB3 and FABP1 expressions, while GABRP and THBS4 were negatively correlated. The macrophages M0 infiltration was positively correlated with both TRIB3 and FABP1 expressions.

**Conclusion:** The protein expressions of TRIB3 and FABP1 gradually decreased with the gastric disease progress, and was positively correlated. *Hp* infection may reduce the protein expression of TRIB3 and FABP1. Combing TRIB3 and FABP1 expressions can improve the diagnostic efficiency for EGC. Either a high expression of TRIB3 or FABP1 indicates a better prognosis for GC. TRIB3 and FABP1 may interact with CEACAM5, PRAP1, GABRP and THBS4, and affect tumor immune microenvironment by regulating immune cells, and participate in the development and progression of GC.

## 1 Background

Gastric cancer (GC) is one of the most common malignant tumors of the digestive tract and ranked fourth in morbidity and second in mortality among all tumor types (Topi et al., 2020). Generally, GC arises from a series of diseases such as chronic superficial gastritis (GS), intestinal metaplasia atrophic gastritis (IM-GA), and dysplasia (Valenzuela et al., 2015). With the rapid progress in molecular biology, more and more evidence has shown that some crucial genes might be involved in this multi-stage process (Joo et al., 2001). Therefore, it is very necessary to identify key molecules or prognostic biomarkers during the dynamic development of gastric diseases.

TRIB3, which belongs to the tribbles pseudokinase family, is encoded by the six exons (20p13-p12.2) located on human chromosome 20. It’s a protein of about 65 kDa containing 358 amino acids (Huang et al., 2003; Wu et al., 2003; Kiss-Toth et al., 2004). Studies have shown that TRIB3 might be an oncogene or a tumor suppressor depending on the specific tumor types (Mondal et al., 2016). The overexpression of TRIB3 in lung and colorectal cancer can regulate some important cancer signaling pathways (Wennemers et al., 2011a; Izrailit et al., 2013). *Wennemer et al* found that the high expression of TRIB3 was significantly associated with better prognosis in breast cancer (Wennemers et al., 2011b).

Liver fatty acid binding protein (FABP1) is a soluble protein of 14 kDa. It is widely distributed in the cytoplasm of hepatocytes, but seldom detected in the nucleus or mitochondrial outer membrane (Börchers et al., 1989; Bordewick et al., 1989; Fahimi et al., 1990). FABP1 also presents in many other cell types, such as intestinal cells, renal tubular cells, and alveolar epithelial cells (Ho and Storch, 2001; Haunerland and Spener, 2004; Chmurzynska, 2006; Furuhashi and Hotamisligil, 2008), and plays a key role in the storage and degradation of fatty acids (Georgiadi and Kersten, 2012). It has been demonstrated that FABP1 was downregulated in nearly 10% of hepatocellular carcinomas (Inoue et al., 2014). The low expression of FABP1 in adenoma was related to the occurrence and development of colorectal cancer (Lee et al., 2006). In addition, FABP1 was found significantly downregulated in kidney cancer and its low expression indicated poor disease-free survival (Wu et al., 2020).

Our previous studies have suggested that TRIB3 and FABP1 were differentially expressed in GA vs GC, and they may interact at protein level. These results suggested that they might participate in the progress of gastric diseases (Liu et al., 2020). However, the correlation between TRIB3 and FABP1 remains undefined, and the expression characteristics during gastric carcinogenesis, as well as the clinical value and potential functions of these two proteins, and remain largely unknown.

Therefore, our study detected the protein expression of TRIB3 and FABP1 in GS, IM-GA, early gastric cancer (EGC) and advanced gastric cancer (AGC) by immunohistochemical staining, aimed to clarify their correlation and roles in gastric carcinogenesis, including their associations with clinicopathological features of GC, and their diagnostic and prognostic values. We further explored the potential functions of TRIB3 and FABP1 by bioinformatics. Our study would lay firm foundations for further exploring the underlying mechanism of the involvement of TRIB3 and FABP1 in the initiation and development of GC.

## 2 Materials and Methods

### 2.1 Patients and Tissue Specimens

A total of 479 patients who were diagnosed in the First Affiliated Hospital of China Medical University from December 2012 to December 2018 were enrolled, including 89 patients with GS, 102 patients with IM-GA, 144 patients with EGC and 144 patients with AGC. Tumor tissues and their corresponding adjacent IM and distal normal (NO) tissue specimens were obtained from 47 AGC patients. The *Helicobacter pylori* (*Hp*) infection status was detected using ASSURE *Hp* rapid test (MP Diagnostics^TM^). Histology classification was determined according to the updated Sydney System for gastritis (Dixon et al., 1996). The diagnosis of GC was based on the World Health Organization criteria (4th edition, 2010). The TNM staging of GC was determined in accordance with the NCCN Clinical Practice Guidelines (2nd Edition, 2018).

The follow-up was continued until November 2019 and the time of it ranged from 11 months to 83 months. Totally, 112 cases were included for prognosis analysis and 58 of them died at data cut-off date, with a median survival time of 30.1 months. Approval was obtained from the Research Medical Ethics Committee of the First Affiliated Hospital of China Medical University, and each subject provided written informed consent.

### 2.2 Immunohistochemistry Staining

IHC was performed mainly as previously described (Yang et al., 2018). 4-µm-thick tissues were mounted in poly-l-lysine-coated glass slides and baked overnight at 65°C. After deparaffinization with xylene and hydration with graded ethanol, tissue sections were heated in EDTA buffer for 20 min for antigen retrieval. The 10% normal goat serum was used for incubation for 15 min to reduce non-specific binding. Then sections were then incubated with primary antibody for 60 min at room temperature (24–27°C) and secondary antibody for 10 min. At last, the slides were stained with DAB (DAB-1031, Maxim Inc., Fujian, China) and counterstained with hematoxylin. The anti-TRIB3 (ab-137526, 1:1,000 dilution; Abcam, Cambridge, United Kingdom) and anti-FABP1 (ab-171739, 1:4,000 dilution; Abcam, Cambridge, United Kingdom) antibodies were used in our experiment.

### 2.3 Evaluation of IHC Staining

Two pathologists individually evaluated the protein expression of TRIB3 and FABP1 in different tissues through multiplying the staining intensity by the proportion of stained cells to get the final immunoreactivity score (IS) without knowing their clinical information. The staining intensity of cells was classified into 0 (no staining), 1 (light brown), 2 (brown staining), and 3 (heavy brown staining); the proportion of stained cells were recorded as 0 (0–5%), 1 (6–25%), 2 (26–50%), 3 (51–75%), and 4 (76–100%). We used IS = 0 (the median of immunohistochemical staining scores) to distinguish between the negative and positive expression of TRIB3 and FABP1.

### 2.4 Analysis of Biological Functions

R software (R 4.0.3) was applied for co-expression analysis utilizing TCGA data normalized by the log2 (TPM (Transcripts per million) +1] transformation. DAVID (https://david.ncifcrf.gov/home.jsp) website was chosen for GO and KEGG pathway analysis. Cibersort was applied to estimate the percentage of different infiltrative immunocytes of each tumor sample of TCGA STAD dataset.

### 2.5 Statistical Analysis

All statistical analyses were performed using SPSS software (version 26.0) or R software (R 4.0.3). χ2 test was applied to assess the differences in age, gender and *Hp* infection between groups. Non-parametric test was applied to evaluate the differential expression of TRIB3 and FABP1 among different gastric diseases and the relationship of their expression with clinicopathological parameters. Paired non-parametric test was selected for comparing the expression of TRIB3 and FABP1 among GC, adjacent IM-GA, and distal NO of the same person. The relationship between TRIB3 and FABP1 protein expressions was assessed by Spearman correlation analysis. Receiver operating characteristic (ROC) curves were used to evaluate the diagnostic values of these two proteins. Survival analysis was evaluated by univariate (Log-rank) and multivariate (Cox model) analysis. The *p*-value < 0.05 was considered statistically significant.

## 3 Results

### 3.1 The Protein Expression of TRIB3 and FABP1 in Different Gastric Diseases

The baseline clinical characteristics of GS, IM-GA, EGC, and AGC patients were listed in Table 1. We analyzed the differential expression of TRIB3 and FABP1 in different disease groups as well as in the cancer and adjacent tissues.

**TABLE 1:** Clinicopathological parameters of GS, IM-GA, EGC, AGC, and survival in AGC.

Characteristics	Categories				*P*	Cases of events	MST	*P*
	GS	IM-GA	EGC	AGC				
Gender					<**0.001**			0.146
Male	42	53	97	106		41	44	
Female	47	49	47	38		17	29	
Age (years)					<**0.001**			0.910
<60	62	39	53	59		24	42	
≥60	27	63	91	85		34	39	
*Hp* infection^a^					**0.004**			0.913
(-)	33	9	18	31		15	46	
(+)	41	49	29	70		33	39	
Tumor location^a^								0.082
Cardia/Body				40		21	31	
Angle/Antrum				96		31	53	
Borrmann type^a^								**0.001**
Ⅰ				2		1	46	
Ⅱ				21		3	No reached	
Ⅲ				79		27	44	
Ⅳ				40		27	28	
Differentiation degree^a^								**0.016**
Poor/Mucinous				108		51	31	
Well/Moderate				33		6	No reached	
TNM stage1								<**0.001**
Ⅰ-Ⅱ				36		4	No reached	
Ⅲ-Ⅳ				106		53	29	
Invasive extent								<**0.001**
T1-3				46		5	No reached	
T4				98		53	24	
Lymph node metastasis^a^								<**0.001**
(-)				39		5	No reached	
(+)				104		53	25	
Distant metastasis								0.138
(-)				142		58	No reached	
(+)				2		0	No reached	
Perineural invasion^a^								**0.002**
(-)				22		2	No reached	
(+)				119		56	30	
Maximum diameter (cm)^a^								**0.045**
<4				22		3	No reached	
≥4				121		54	39	
Growth pattern								0.055
Nested/cloddy				25		7	No reached	
Infiltrative				119		51	39	
Vessel carcinoma embolus								0.059
(-)				53		17	53	
(+)				91		41	31	
Extranodal tumor implantation^a^								<**0.001**
(-)				127		49	42	
(+)				10		9	6	

The bold values: *p* < 0.05.

a Incomplete information.

#### 3.1.1 TRIB3 Protein Expression in Different Gastric Diseases

**FIGURE 1:**
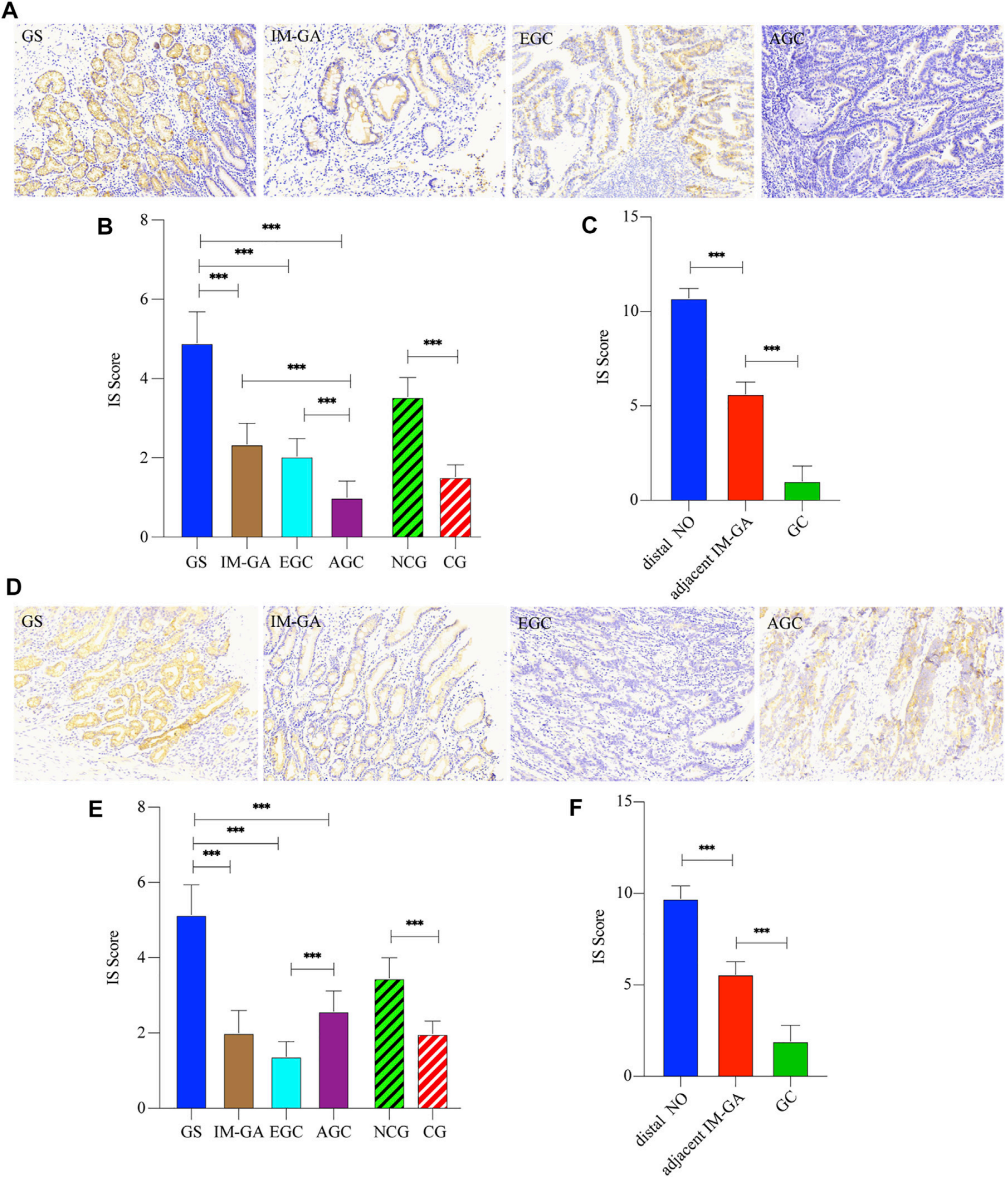
The protein expressions of TRIB3 **(A–C)** and FABP1 **(D–F)** in different gastric tissues as well as in AGC and non-tumor adjacent tissue. Magnification ×200.

The comparison of TRIB3 expression between different gastric diseases was shown in Figure 1. TRIB3 was mainly expressed in the epithelial cytoplasm. Representative staining images were shown in Figure 1A. Compared with GS, TRIB3 expression in the IM-GA, EGC, and AGC groups was significantly decreased (*p* < 0.001, *p* < 0.001, and *p* < 0.001, respectively); compared with IM-GA, TRIB3 expression was decreased in EGC and AGC (*p* < 0.001), though the difference in IM-GA vs EGC was not significant (*p* = 0.174). Specifically, TRIB3 expression in the AGC group was significantly lower than that in the EGC group (*p* < 0.001). Besides, the expression of TRIB3 protein in EGC + AGC group (Cancer group, CG) was significantly lower than that in GS + IM-GA group (Non cancer group, NCG) (*p* < 0.001). Overall, the protein expression trends of TRIB3 among different gastric disease were GS > IM-GA > EGC > AGC and NCG > CG (Figure 1B). Furthermore, the expression of TRIB3 showed a significant decrease from distal NO tissue, adjacent IM-GA to GC in the same patients (*p* < 0.001) (Figure 1C).

#### 3.1.2 FABP1 Protein Expression in Different Gastric Disease

The comparison of FABP1 expression between different gastric diseases was shown in Figure 1. FABP1 was also mainly expressed in the epithelial cytoplasm. Representative staining images were shown in Figure 1D. The expression pattern of FABP1 was similar with that of TRIB3 from GS, IM-GA to EGC (*p* < 0.001, *p* < 0.001, and *p* < 0.001, respectively). However, compared with EGC group, FABP1 expression was significantly increased in AGC group. Similarly, the protein expression of FABP1 in CG was significantly lower than that in NCG (*p* < 0.001). Overall, the protein expression trends of FABP1 were GS > IM-GA > EGC, EGC < AGC, and NCG > CG. (Figure 1E). Similar to TRIB3, the protein expression of FABP1 showed a significant decrease from distal NO tissue, and adjacent IM-GA to GC in the same patients (*p* < 0.001) (Figure 1F).

### 3.2 The Relation of the TRIB3 and FABP1 Expression With Clinical Parameters

We separately assessed the association of the protein expression of TRIB3 and FABP1 with clinical parameters, such as gender, age, *Hp* infection status, differentiation degree, and TNM stage, etc.

#### 3.2.1 TRIB3 Protein Expression and Clinical Parameters

**FIGURE 2:**
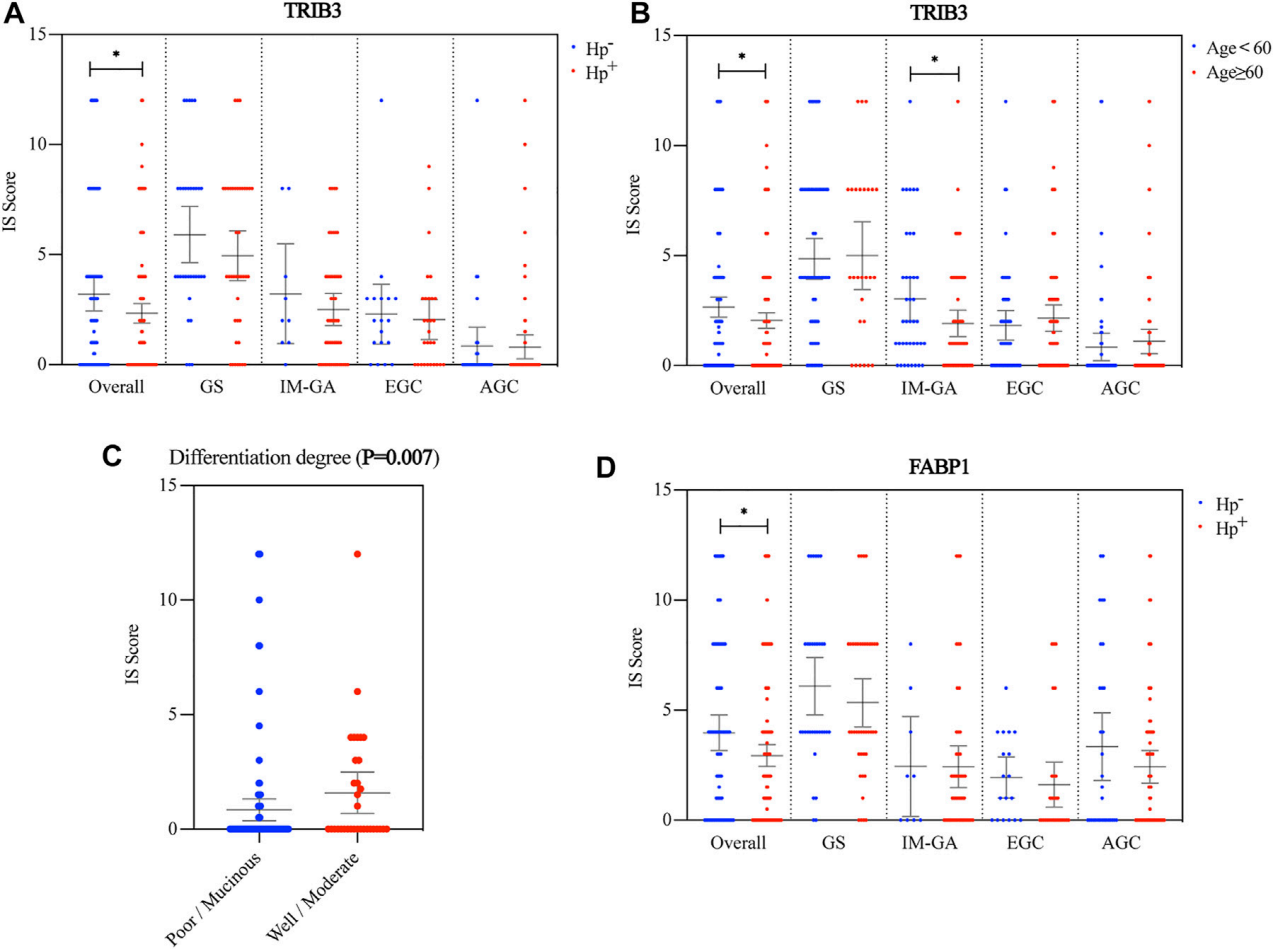
Association between the protein expressions of TRIB3 and FABP1 and clinicopathological parameters. TRIB3 was correlated with *Hp* infection status **(A)** and age **(B)**. Besides, it was significantly correlated with the differentiation degree in AGC **(C)**. FABP1 was also correlated with *Hp* infection status **(D)**.

TRIB3 protein expression in the *Hp* ^+^ group were significantly lower than that in the *Hp* ^−^ group (*p* = 0.038). This lower expression was also observed in patients with GS, IM-GA or EGC though without significance (*p* = 0.285, *p* = 0.428, and *p* = 0.630, respectively) (Figure 2A). We also found that among the overall or the IM-GA patients, the expression of TRIB3 was significantly different in patients with age ≥60 years and those with age <60 (*p* = 0.029, *p* = 0.049, respectively) (Figure 2B). Besides, the AGC patients with well differentiated/mucinous histology had significantly higher TRIB3 expression than those with well/moderate differentiated histology (*p* = 0.007) (Figure 2C). Other clinicopathological parameters didn’t indicate the significantly differential expression of TRIB3 (Supplementary Figure S1).

#### 3.2.2 FABP1 Protein Expression and Clinical Parameters

Consistent with TRIB3, FABP1 showed significantly lower expression in the *Hp* ^+^ group than in the *Hp* ^−^ group (*p* = 0.031). Likewise, this lower expression was observed in patients with GS, IM-GA or EGC though without significance (*p* = 0.320, *p* = 0.193, and *p* = 0.600, respectively) (Figure 2D). Other clinicopathological parameters didn’t indicate the significantly differential expression of FABP1 (Supplementary Figure S2).

### 3.3 The Diagnostic/Prognostic Value of TRIB3 and FABP1 for GC

We evaluated the diagnostic value of TRIB3 and FABP1 protein expressions, separately or combined, for GC and EGC. Their prognostic value in AGC, separately or combined, and was also analyzed in this study.

#### 3.3.1 The Diagnostic/Prognostic Value of TRIB3 for GC

**FIGURE 3:**
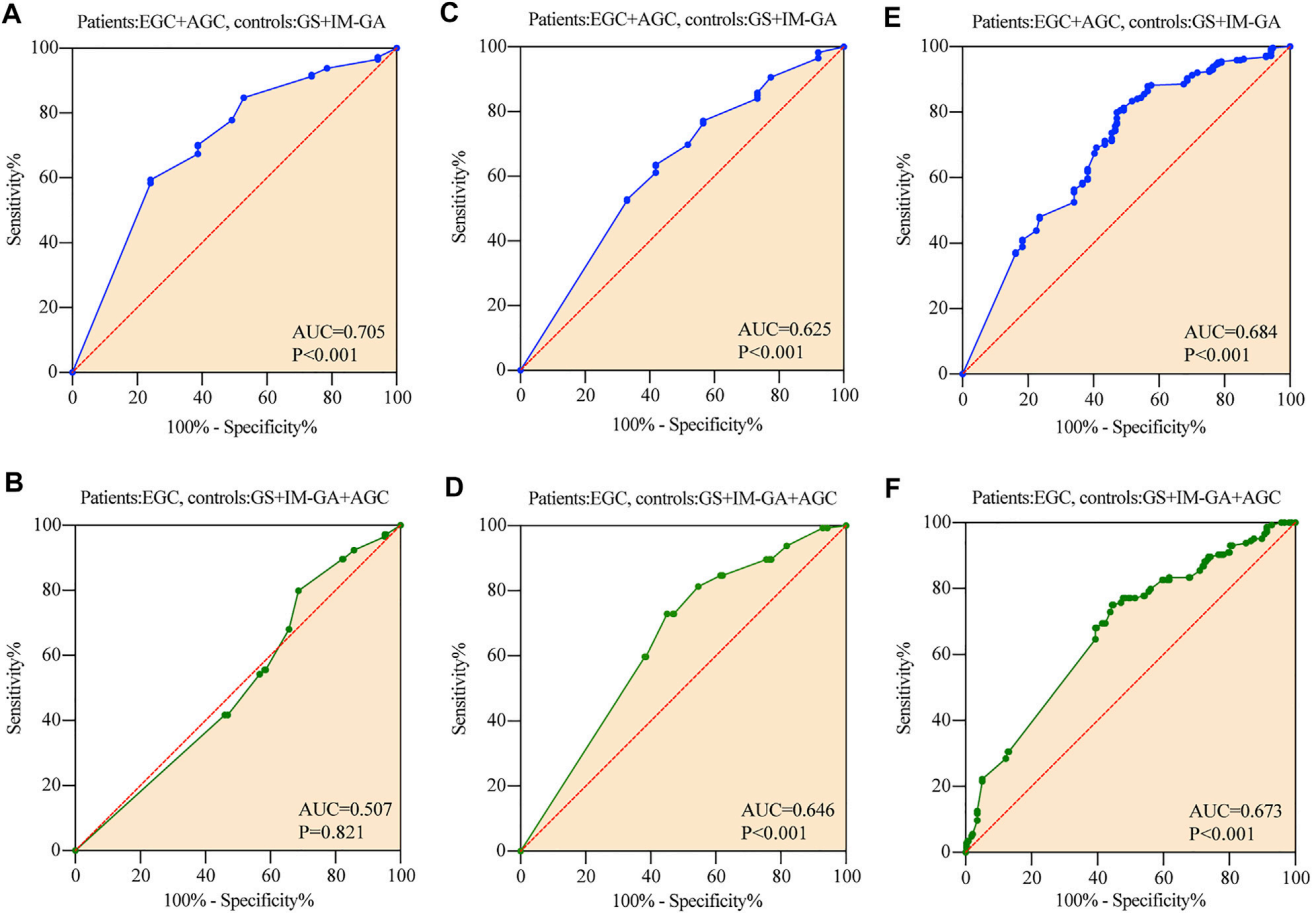
The diagnostic value of TRIB3 and FABP1 for GC. TRIB3 had significant diagnostic value for GC **(A)**, but had no diagnostic value for EGC **(B)**. FABP1 had significant diagnostic value for both GC **(C)** and EGC **(D)**. Combined TRIB3 with FABP1 had significant diagnostic value for both GC **(E)** and EGC **(F)**.

The ROC results showed that the expression of TRIB3 had significant diagnostic value for GC (AUC = 0.705, *p* < 0.001), but had no diagnostic value for EGC (AUC = 0.507, *p* = 0.821) (Figures 3A,B).

**FIGURE 4:**
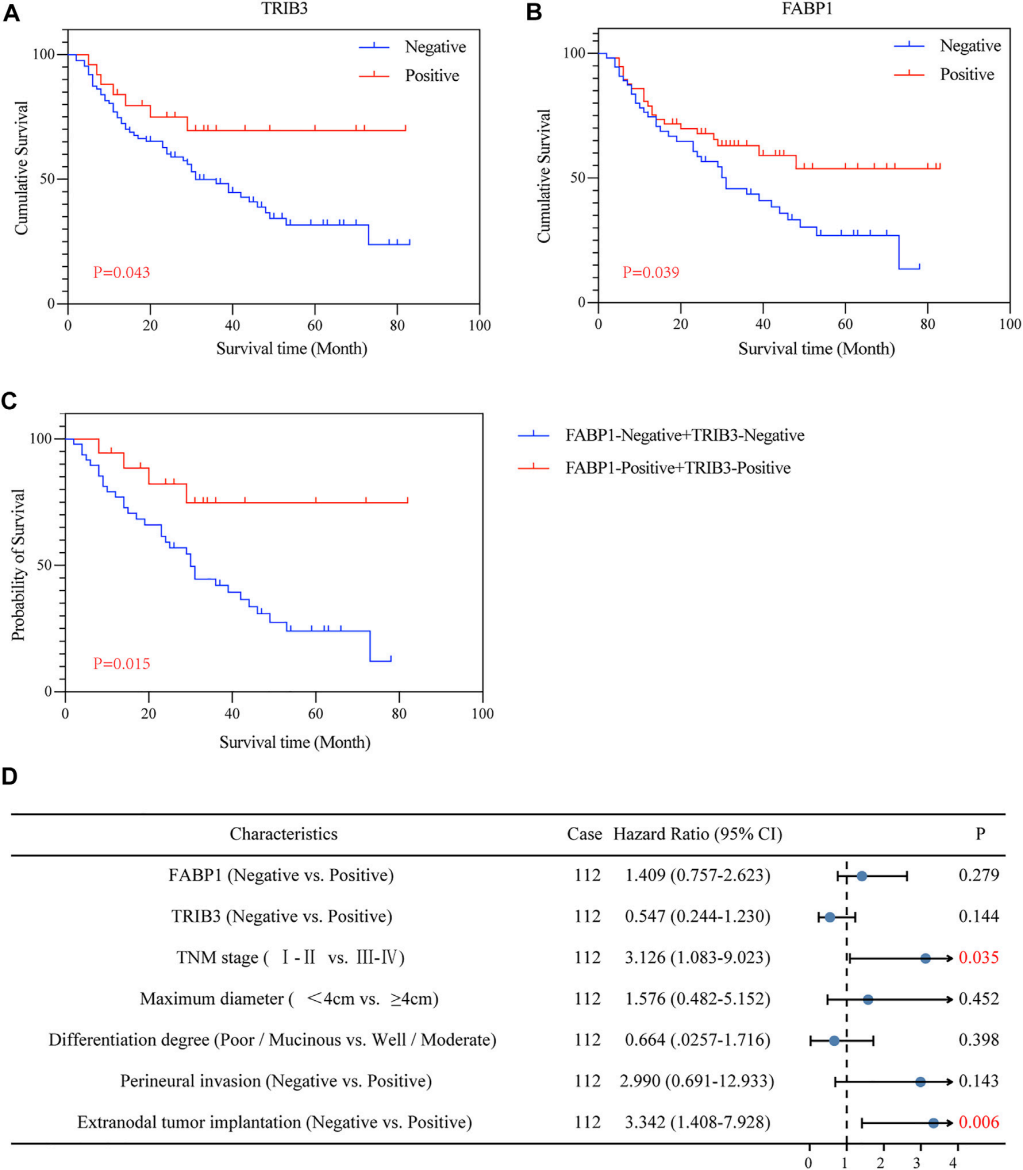
The prognostic value of TRIB3 and FABP1 for AGC. The protein expressions of TRIB3 **(A)** and FABP1 **(B)** tended to have better survival in comparison to those negative. Besides, patients with both positive expression of TRIB3 and FABP1 tended to have superior survival time compared with the both negative patients **(C)**.

The Kaplan-Meier curve showed that AGC patients positive for TRIB3 expression tended to have better survival in comparison to those negative (*p* = 0.043) (Figure 4A). However, multivariate survival analysis didn’t reveal a prognostic significance considering the expression level of TRIB3 in AGC (*p* = 0.144) (Figure 4D; Table 2).

**TABLE 2:** Correlation between the expression of FABP1 and TRIB3 and survival in GC.

Characteristics	Case	Univariate	Multivariate	
		*P*	Hazard ratio (95% CI)	*P*
FABP1 (Negative vs Positive)	112	**0.039**	1.409 (0.757–2.623)	0.279
TRIB3 (Negative vs Positive)	112	**0.043**	0.547 (0.244–1.230)	0.144
TNM stage (Ⅰ-Ⅱ vs Ⅲ-Ⅳ)	112	<**0.001**	3.126 (1.083–9.023)	**0.035**
Maximum diameter (<4 cm vs ≥4 cm)	112	**0.045**	1.576 (0.482–5.152)	0.452
Differentiation degree (Poor/Mucinous vs Well/Moderate)	112	**0.016**	0.664 (0.0257–1.716)	0.398
Perineural invasion (Negative vs Positive)	112	**0.002**	2.990 (0.691–12.933)	0.143
Extranodal tumor implantation (Negative vs Positive)	112	<**0.001**	3.342 (1.408–7.928)	**0.006**

The bold values: *p* < 0.05.

#### 3.3.2 The Diagnostic/Prognostic Value of FABP1 for GC

The expression of FABP1 had significant diagnostic value for both GC and EGC, with an AUC of 0.646 for EGC (*p* < 0.001), and an AUC of 0.625 for GC (*p* < 0.001) (Figures 3C,D).

Moreover, AGC patients with positive FABP1 expression achieved better prognosis than those with negative FABP1 expression (*p* = 0.039) (Figure 4B); whereas the multivariate survival analysis suggested that FABP1 was not an independent prognostic factor of AGC (*p* = 0.279) (Figure 4D; Table 2).

#### 3.3.3 Combined Diagnostic/Prognostic Value for GC

The value of TRIB3 combined with FABP1 to diagnose GC (AUC = 0.684, *p* < 0.001) was lower than that of TRIB3 alone (Figure 3E). However, the diagnostic efficiency of the combination of these two indicators to diagnose EGC was higher than that of a single indicator (AUC = 0.673, *p* < 0.001) (Figure 3F).

Interestingly, AGC patients with both positive expression of TRIB3 and FABP1 tended to have superior survival time compared with the both negative patients (*p* = 0.015) (Figure 4C).

### 3.4 Significantly Positive Correlation Between the Protein Expression of TRIB3 and FABP1

**FIGURE 5:**
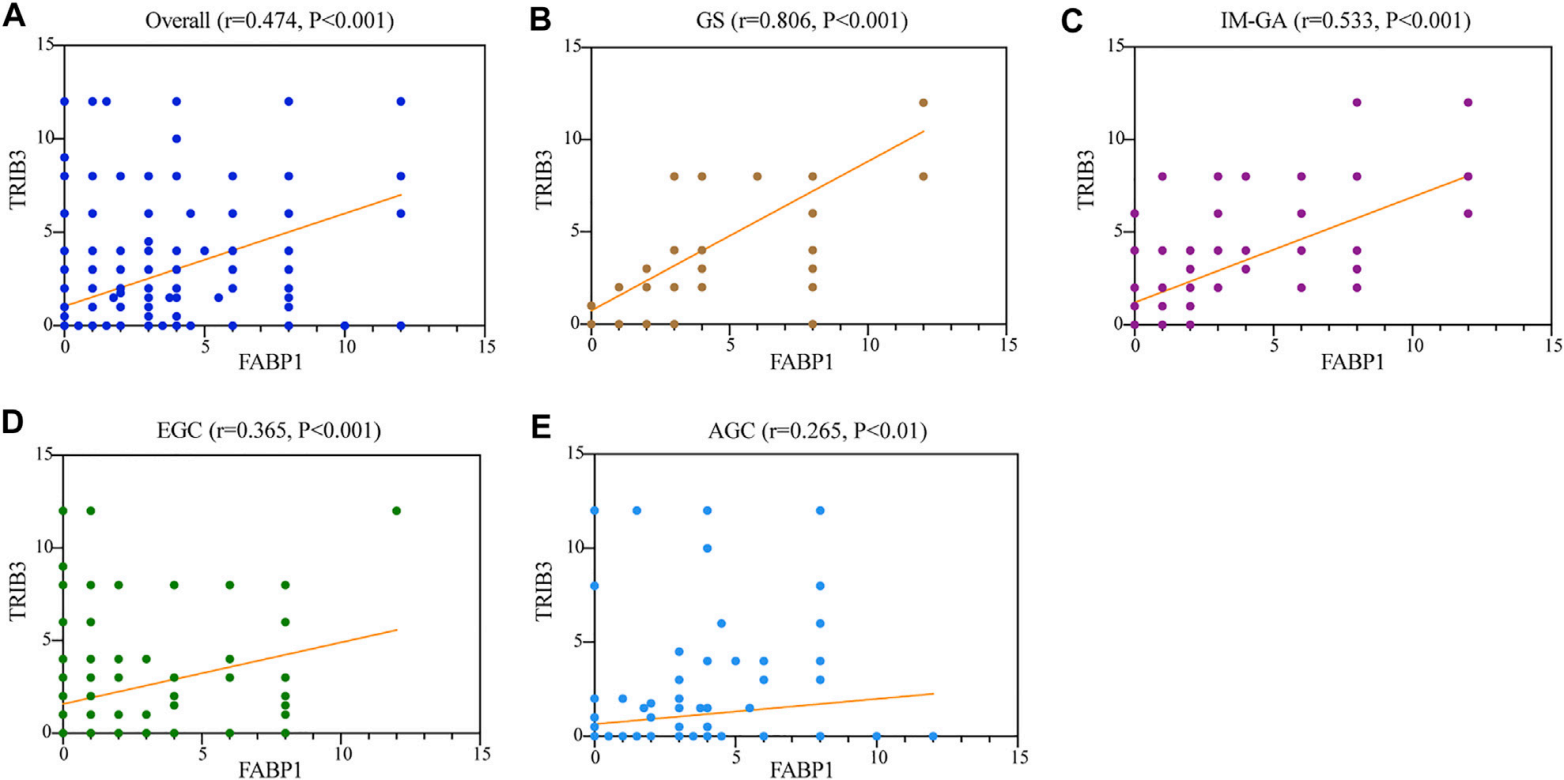
The protein expressions of TRIB3 and FABP1 were significantly positively correlated in the overall patients **(A)**, GS **(B)**, IM-GA **(C)**, EGC **(D)**, and AGC **(E)**.

The spearman correlation analysis was used to evaluate the relationship between TRIB3 and FABP1 protein expressions (Figure 5). The protein expressions of TRIB3 and FABP1 were significantly positively correlated in the overall patients (r = 0.474, *p* < 0.001) (Figure 5A) as well as in each disease group. Interestingly, during the development and progression of gastric disease, and the correlation coefficient gradually decreased (GS: r = 0.806, *p* < 0.001; IM-GA: r = 0.533, *p* < 0.001; EGC: r = 0.365, *p* < 0.001; AGC: r = 0.265, *p* < 0.01) (Figures 5B–E).

### 3.5 Identification of Genes Co-expressed With TRIB3 and FABP1

**FIGURE 6:**
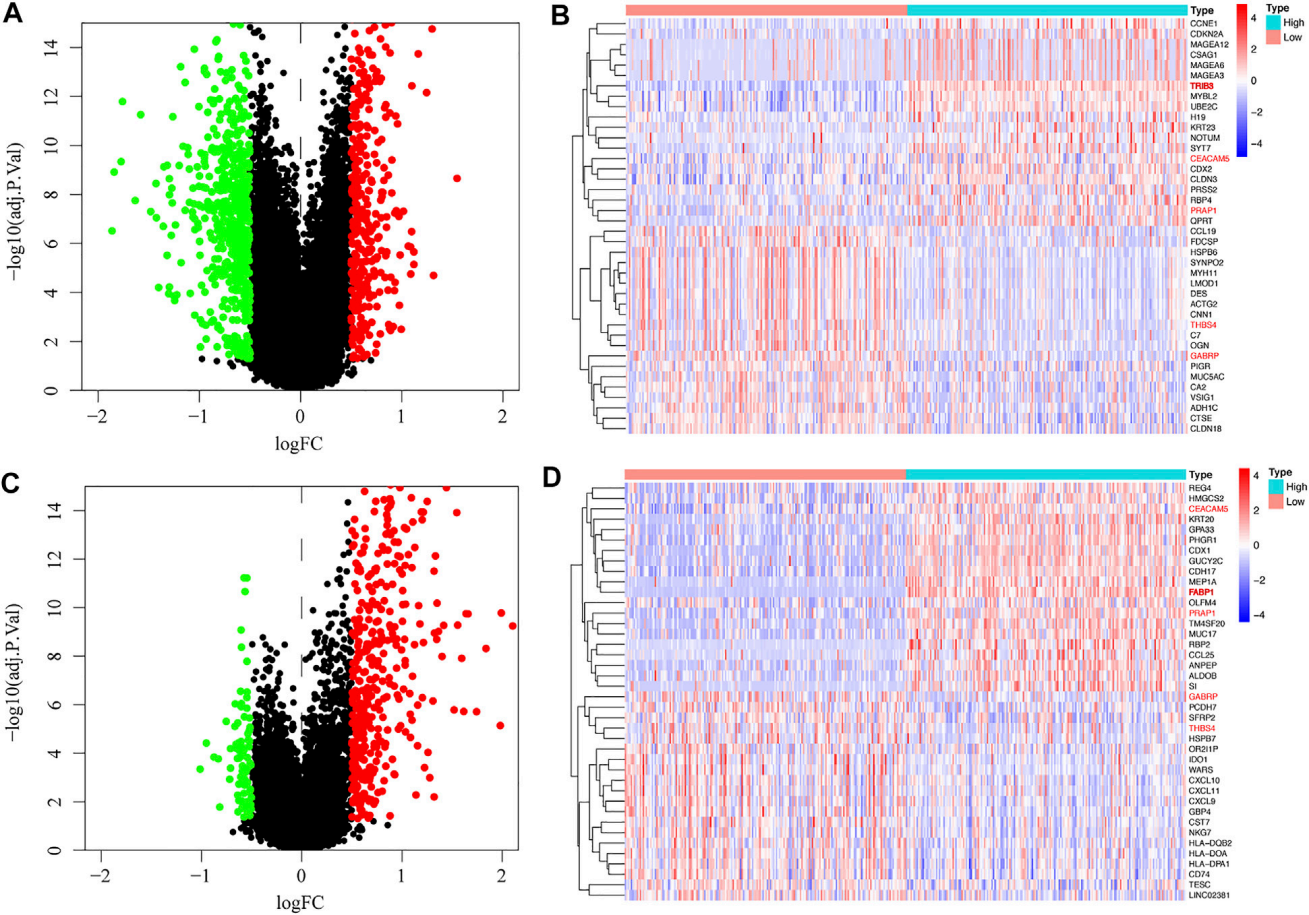
Genes co-expressed with TRIB3 **(A)** and FABP1 **(C)** were searched, with the top 20 positively or negatively regulated genes of TRIB3 **(B)** or FABP1 **(D)** visualized by the heat plot.

We searched for genes co-expressed with TRIB3 (Figure 6A) and FABP1 (Figure 6C) respectively based on |logFC| ≥ 0.5 and adjust *p* value ≤0.05, with the top 20 positively or negatively regulated genes of TRIB3 (Figure 6B) or FABP1 (Figure 6D) visualized by the heat plot. We found that CEACAM5 and PRAP1 were positively correlated with both TRIB3 and FABP1 expressions, while GABRP and THBS4 were negatively correlated with both TRIB3 and FABP1 expressions.

### 3.6 Potential Functions and Pathways of TRIB3 and FABP1 by GO and KEGG Analyses

**FIGURE 7:**
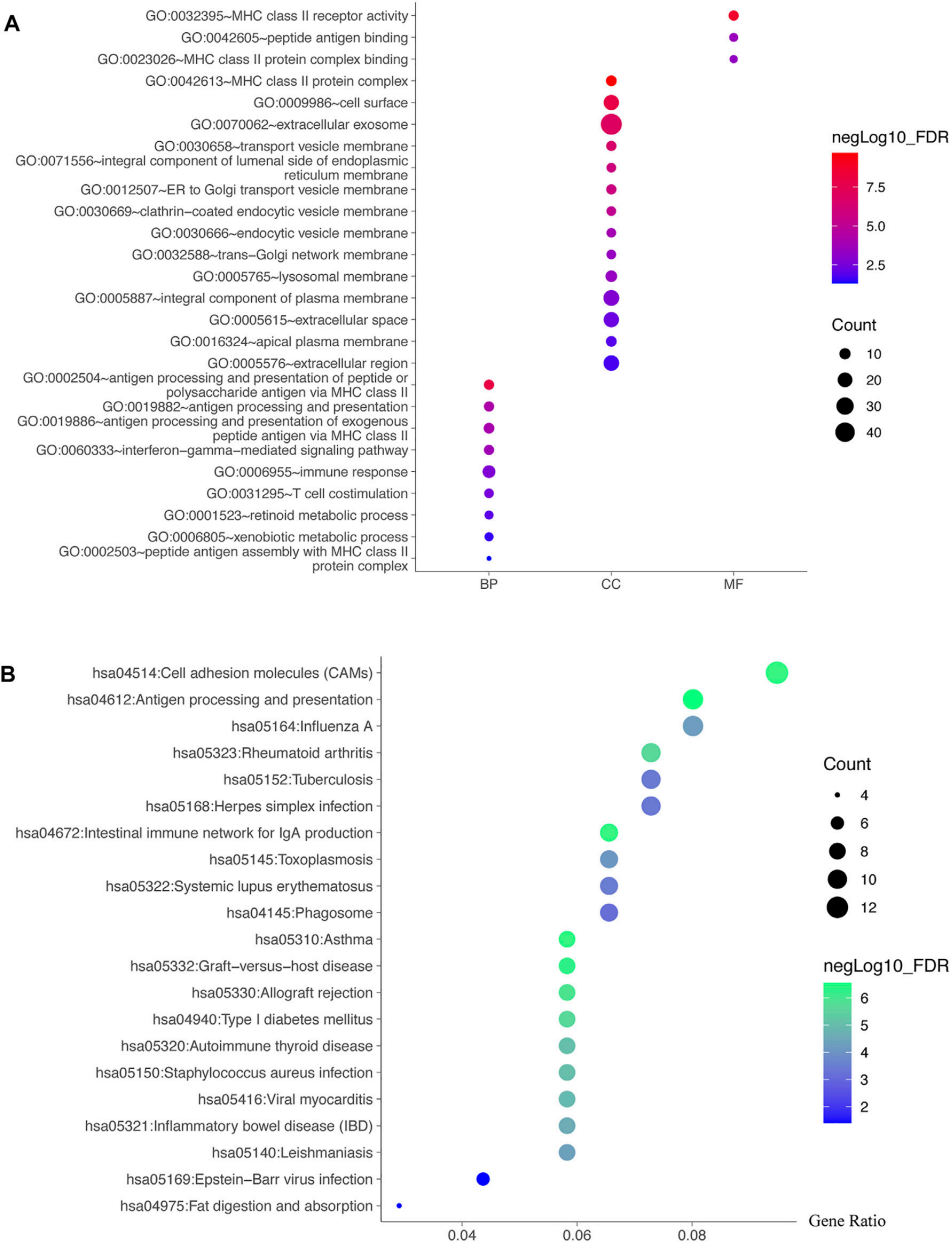
Potential functions and pathways of TRIB3 and FABP1 by GO and KEGG analyses. The genes shared by the two co-expression analyses were used for further GO **(A)** and KEGG **(B)** analyses.

The genes shared by the two co-expression analyses were used for further GO and KEGG analyses. The GO functions enriched in biological processes (BP) were antigen processing and presentation, immune response, and T cell co-stimulation, etc. The significantly enriched cell components (CC) terms were MHC class II protein complex, cell surface and extracellular exosome, etc. The molecular function (MF) significantly related to TRIB3 and FABP1 were MHC class II receptor activity, peptide antigen binding and MHC class II protein complex binding (Figure 7A). Moreover, the KEGG pathway analysis suggested that the shared co-expression genes were mainly enriched in cell adhesion molecules, inflammatory bowel disease and intestinal immune network for IgA production (Figure 7B).

### 3.7 Correlation Between Immune Infiltration and Expression of TRIB3 and FABP1

**FIGURE 8:**
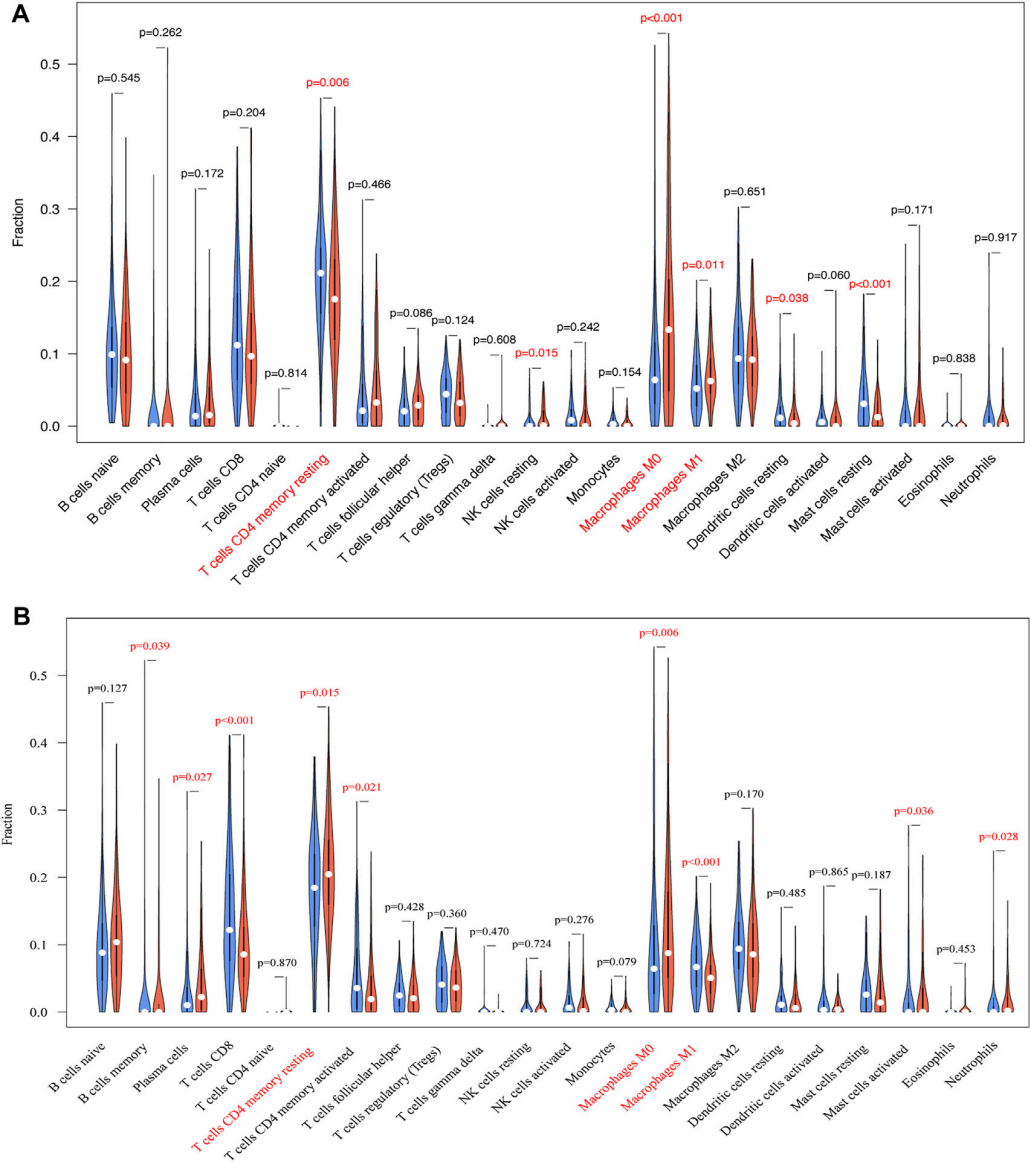
The associations of TRIB3 **(A)** and FABP1 **(B)** with immunocyte infiltrations in GC were further evaluated.

Due to the potential relevance of TRIB3 and FABP1 in immune-related process as suggested by the GO and KEGG analysis, we further evaluated their associations with immunocyte infiltrations in GC. TRIB3 expression was indicative of the infiltrations of T cells CD4 memory resting, NK cell resting, macrophages M0, macrophages M1, dendritic cells activated and mast cells resting (Figure 8A), while FABP1 expression was associated with the infiltrations of B cells memory, plasma cells, T cells CD8, T cells CD4 memory resting, T cells CD4 memory activated, macrophages M0, macrophages M1, and mast cells activated and neutrophils (Figure 8B). Notably, the infiltration of T cells CD4 memory resting was negatively correlated with TRIB3 but positively associated with FABP1 expression, while the infiltration of macrophages M1 was positively correlated with TRIB3 but negatively associated with FABP1 expression. Besides, the macrophages M0 infiltration was positively correlated with both TRIB3 and FABP1 expressions.

## 4 Discussion

In this study, we elucidated the protein expression of TRIB3 and FABP1 in different stages of gastric disease, and identified the correlation between their expressions. Their diagnostic and prognosis values, as well as their associations with clinicopathological parameters were also analyzed. Furthermore, the potential functions of TRIB3 and FABP1 were explored through TCGA and David database. As far as we know, this is the first study assessing the expression trend of TRIB3 and FABP1 in the dynamic process of gastric disease development, and also the first report correlating TRIB3 with FABP1. This study identified candidate diagnostic and prognostic biomarkers for the early detection of GC, and provided evidence and insight for further study revealing the molecular mechanism underlying the GC development and progression.

The occurrence of GC is a multi-stage process that go through GS and IM-GA. As the disease developed, EGC progressed to AGC (Liu et al., 2020). Our results showed that during this multi-stage process, the expression trend of TRIB3 and FABP1 protein was GS > IM-GA > EGC. Besides, TRIB3 and FABP1 was significantly lower expressed in GC tissues than in adjacent IM-GA or distal NO tissue. These results indicated that the expression of TRIB3 and FABP1 was downregulated when GC occurred. Some relevant studies have reported that TRIB3 played a key role in the anti-cancer activity of cannabinoids, and the gene inactivation of TRIB3 promoted the occurrence of cancer (Vara et al., 2013). *Supriya Srivastava et al.* observed that the protein expression of FABP1 in esophageal carcinoma was significantly lower than that in esophageal dysplasia, which could be used as a key auxiliary diagnostic index to determine the status of disease progress (Srivastava et al., 2017). *Takeaki Hashimoto et al.* reported that FABP1 protein was highly expressed in IM but lacking in most GC patients (Hashimoto et al., 2004). Specifically, we found that compared with EGC, the expression of TRIB3 protein continued to decrease in AGC, and while the expression of FABP1 was abnormally increased. The above results showed that TRIB3 expression was successively decreased during the process of GC initiation and development; whereas FABP1 was expressed in a two-stage pattern: its expression was decreased at first, and then increased during the progress of a disease. Previous studies based on several cancer cells and animal models had found that the inactivation of TRIB3 regulated Akt pathway and enhanced tumor development (Salazar et al., 2015), while FABP1 was significantly downregulated in some subtypes of colorectal cancer due to the influence of immune microenvironment (Wood et al., 2017). These implied the important role of TRIB3 and FABP1 in GC development. Similar to FABP1, the two-stage expression pattern of CDX2 and SOX9 during the initiation and development of GC was also reported previously (Uozaki et al., 2011; Sun et al., 2012), suggesting that these molecules might play different roles in different stages of gastric disease progression.

It’s commonly accepted that *Hp* infection was closely related to the occurrence of GC (Uozaki et al., 2011; Liu et al., 2020). We observed that *Hp* infection was significantly associated with the decreased expression of TRIB3 and FABP1, indicating that the decreased expression of TRIB3 and FABP1 in GC lesions was partly due to the *Hp* infection. A prior meta-analysis revealed that *Hp* infection probably caused hypermethylation of CDH1, which was significantly associated with the risk of GC (Zeng et al., 2015). And TRIB3 was reported to be ubiquitinated by CDH1 and then degraded (Ohoka et al., 2010). Based on these studies, we speculate that the infection of *Hp* may regulate TRIB3 expression through the ubiquitination-proteasome pathway mediated degradation. In addition, studies have reported that antibacterial drugs could alleviate the decrease in FABP1 expression (Brown et al., 2016). Furthermore, we explored the relationship between the expressions of TRIB3 and FABP1 and their associations with clinicopathological parameters of AGC. AGCs that were poor differentiated/mucinous presented lower TRIB3 expressions than the well/moderate differentiated ones. Our result was consistent with the previous reports that TRIB3 was significantly correlated with the degree of bone marrow differentiation in patients with acute promyelocytic leukemia (Li et al., 2017). Since differentiation is a key factor reflecting the invasiveness of tumors, the low expression of TRIB3 may indicate a higher malignant degree of GC.

In addition, we found that the expression of TRIB3 were of diagnostic value for GC, while FABP1 were of diagnostic value for both GC and EGC, and as suggested by the previous research results in renal cancer and breast cancer (Wennemers et al., 2011a; Wu et al., 2020). Moreover, combining these two proteins was proved to improve the diagnostic efficiency for EGC. These results suggested that TRIB3 can be used as a diagnostic biomarker for GC, while FABP1 is more effective in diagnosing EGC. The diagnostic efficiency for EGC was higher when combining TRIB3 and FABP1.

Studies have shown that the high expression of TRIB3 in breast cancer and oral squamous cell carcinoma indicated a better prognosis (Wennemers et al., 2011a; Zhang et al., 2011), but whether it can affect GC prognosis has not been confirmed. *Takeaki Hashimoto et al.* reported that the expression level of FABP1 was not associated with GC prognosis (Hashimoto et al., 2004); while another study revealed that it was a reliable prognostic indicator of GC (Satoh et al., 2012). Until now, no definite conclusion formed on the relationship between the expression of FABP1 and the prognosis of GC patients. Our study evaluated the prognostic roles of TRIB3 and FABP1 in GC by univariate and multivariate survival analyses. And the results suggested that a higher expression of TRIB3 or FABP1 could indicate a better prognosis of GC, but it was not significant in multivariate survival analyses. It had been demonstrated that TRIB3 was involved in the normal death process of tumor cells (Ohoka et al., 2005; Örd et al., 2007; Salazar et al., 2009). The high protein expression of TRIB3 could inhibit the ability of tumor immune apoptosis, cause tumor cell death and prolong the survival of patients (Wennemers et al., 2011b). Besides, our results were consistent with those of *Yumiko Satoh et al.*, and which indicated that FABP1 was a reliable prognostic indicator of GC (Satoh et al., 2012).

We also evaluated the correlation between the expressions of TRIB3 and FABP1. The expressions of TRIB3 and FABP1 were positively correlated regardless of the diseases the patients harbored. Of note, the correlation coefficients between TRIB3 and FABP1 expressions gradually weakened as the gastric disease progressed from GS to GC, suggesting that there might be direct or indirect interactions between TRIB3 and FABP1 and the strength of this interaction can be regulated during GC development. Interestingly, the correlation between TRIB3 and FABP1 was stronger in gastric tissues that tend to be normal, and which was worthy of further study.

To further explore the potential functions of TRIB3 and FABP1, we conducted bioinformatics mining. Co-expression and correlation analyses suggested that CEACAM5 and PRAP1 expressions were positively correlated with both TRIB3 and FABP1 expressions, while GABRP and THBS4 expressions were negatively correlated with them. CEACAM5 was a human epidermal cell adhesion molecule, which could interact with *Hp* adhesion protein HopQ and transfer CagA protein to the host gastric epithelial cells, thus triggering inflammation (Xia et al., 2019). PRAP1 was a proline-rich acidic protein that could down-regulate MAD1 and inhibit mitotic checkpoint signaling in hepatocellular carcinoma (Sze et al., 2014). GABRP has been reported to play an immunomodulatory role in pancreatic cancer in a neurotransmitter independent manner, promoting macrophage infiltration by inducing the expression of CXCL5 and CCL20, and thereby affecting tumor growth and metastasis (Jiang et al., 2019). Studies demonstrated that THBS4, an extracellular glycoprotein, was involved in wound healing and tissue remodeling. It was found to be expressed on fibroblasts in the microenvironment of GC and was related to the metastasis of cancer cells (Kuroda et al., 2019). Our findings suggested that they might be active molecules involved in the interaction between TRIB3 and FABP1.

GO and KEGG enrichment analyses revealed the possible role of TRIB3 and FABP1 in immune related process, and further immune infiltration analysis elucidated that TRIB3 and FABP1 may play a role in the inhibition of CD4^+^ T cells infiltration and the differentiation of macrophages. Existing studies have found that TRIB3 participated in the differentiation of CD4^+^ T cells and played an important role in the apoptosis of macrophages induced by oxidized low density lipoprotein (Shang et al., 2009; Kiuchi et al., 2021). FABP1 had not been reported to be related to CD4^+^ T cells, but it could affect the function of tumor-associated macrophages by participating in tumor internal metabolic pathways, and thereby exerting anti-tumor effects (Xu et al., 2020). The above results suggest that TRIB3 and FABP1 may affect the tumor immune microenvironment by acting on immune cells, and then participate in the process of GC development.

However, there are still some limitations in our study. Although we have tried to reveal the functional role of TRIB3 and FABP1 by exploring co-expressed genes and immune infiltration, there is currently no direct evidence support the hypothesis, and which needs to be further investigated.

## 5 Conclusion

In conclusion, the protein expressions of TRIB3 and FABP1 gradually decreased with the gastric disease progress, and was positively correlated. *Hp* infection may reduce the protein expression of TRIB3 and FABP1. In addition, the low expression of TRIB3 protein is associated with the malignant biological behavior of GC. Combing TRIB3 and FABP1 expressions can improve the diagnostic efficiency for EGC. Either a high expression of TRIB3 or FABP1 indicates a better prognosis for GC. Bioinformatics analysis showed that TRIB3 and FABP1 may interact with CEACAM5, PRAP1, GABRP, and THBS4, and affect tumor immune microenvironment by regulating immune cells, and participate in the development and progression of GC.

## Data Availability

The original contributions presented in the study are included in the article/Supplementary Material, further inquiries can be directed to the corresponding authors.

## Abbreviations

AGC, advanced gastric cancer; BP, biological processes; CC, cell components; CG, Cancer group; EGC, early gastric cancer; FABP1, Liver fatty acid binding protein; GC, gastric cancer; GS, superficial gastritis; Hp, Helicobacter pylori; IHC, immunohistochemistry; IM-GA, intestinal metaplasia atrophic gastritis; IS, immunoreactivity score; MF, molecular function; NCG, Non cancer group; NO, normal; ROC, Receiver operating characteristic; TCGA, The Cancer Genome Atlas; TRIB3, Tribbles Pseudokinase 3.
