# Supplementary material for: The Involvement of TRIB3 and FABP1 and Their Potential Functions in the Dynamic Process of Gastric Cancer
Source: Front Mol Biosci. 2021 Dec 9;8:790433. doi: 10.3389/fmolb.2021.790433 (PMC8696077; doi:10.3389/fmolb.2021.790433)
Supplement: Supplementary file 1 [file DataSheet3.DOCX]

**
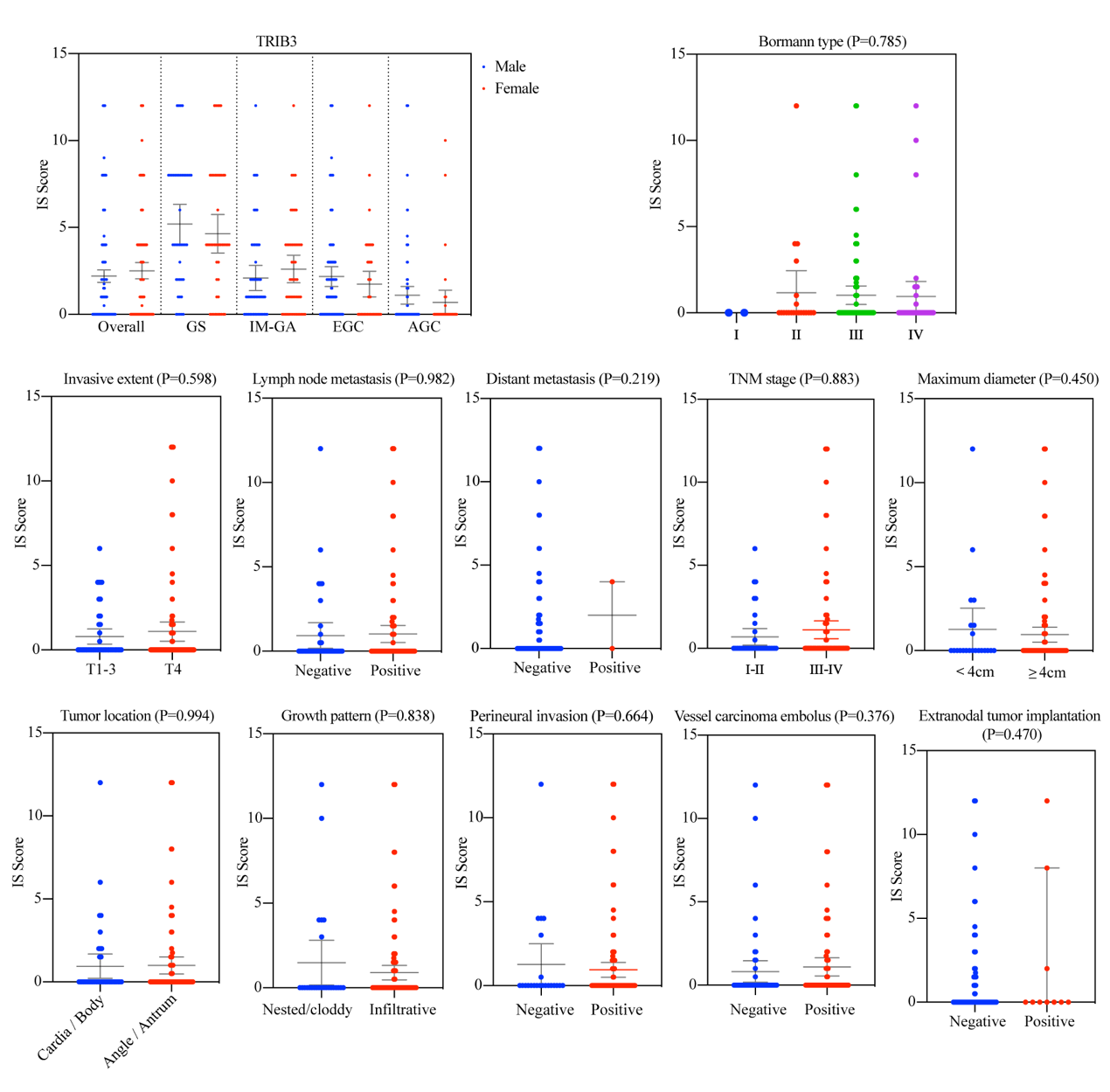
**

**Figure S1. Other clinicopathological parameters didn’t indicate the significantly differential expression of TRIB3.**

**
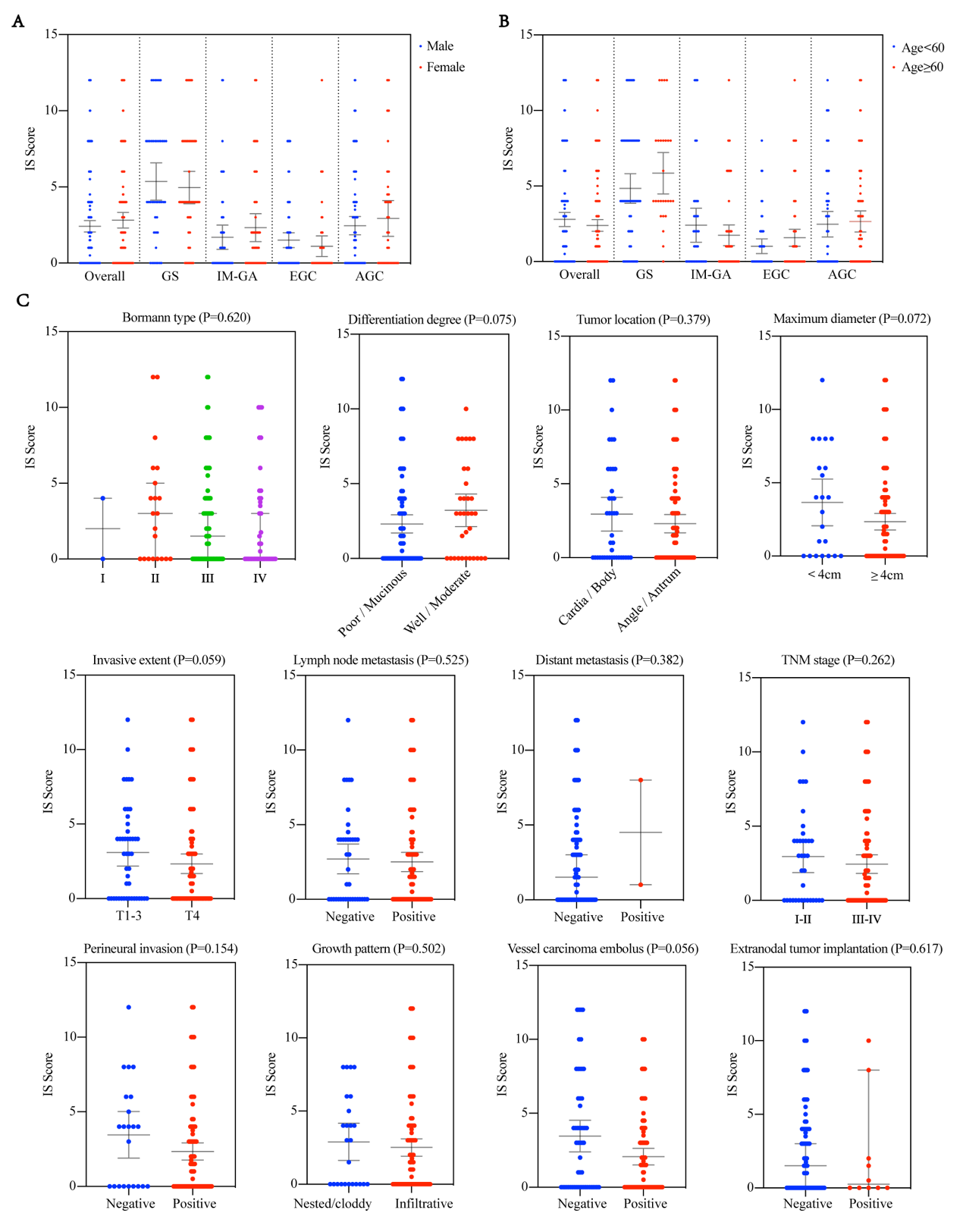
**

**Figure S2. Other clinicopathological parameters didn’t indicate the significantly differential expression of FABP1.**
