# Supplementary figures and images for: The Involvement of TRIB3 and FABP1 and Their Potential Functions in the Dynamic Process of Gastric Cancer
Source: Front Mol Biosci. 2021 Dec 9;8:790433. doi: 10.3389/fmolb.2021.790433 (PMC8696077; doi:10.3389/fmolb.2021.790433)

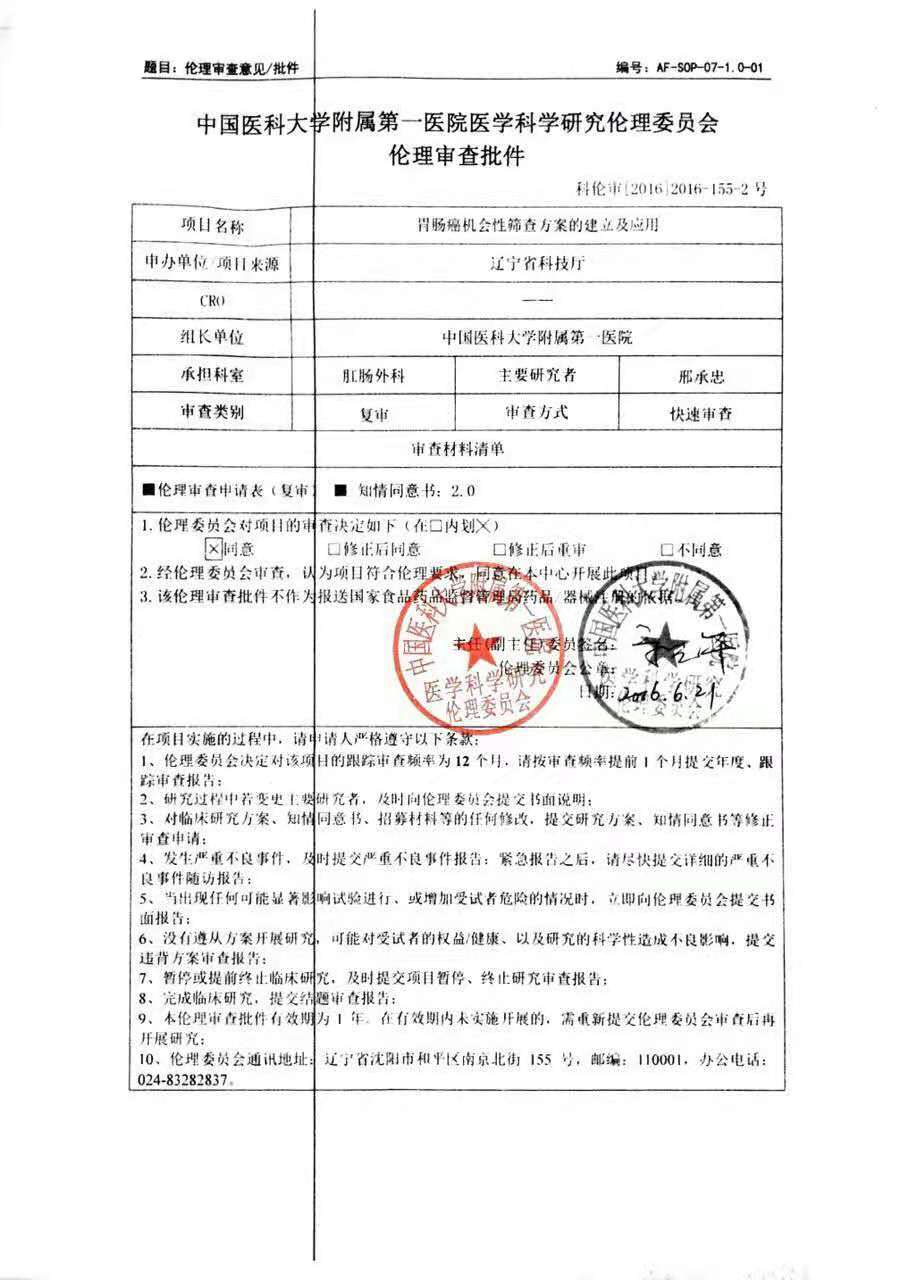

Supplement: Supplementary file 2 [file DataSheet2.ZIP › Original ethical approval/Original ethical approval.jpeg]
