# Supplementary material for: The Involvement of TRIB3 and FABP1 and Their Potential Functions in the Dynamic Process of Gastric Cancer
Source: Front Mol Biosci. 2021 Dec 9;8:790433. doi: 10.3389/fmolb.2021.790433 (PMC8696077; doi:10.3389/fmolb.2021.790433)
Supplement: Supplementary file 2 [file DataSheet2.ZIP › Original ethical approval/Original ethical approval.doc]

**Ethics Committee of Medical Science Research in the First Hospital of China Medical University**

**Approval of Ethics Review**

Review No. {[2016]2016-155-2}

| Project | Establishment and application of opportunistic screening program for gastrointestinal cancer | | |
| --- | --- | --- | --- |
| Sponsor/project source | Science and Technology Program of Liaoning Province | | |
| CRO | — | | |
| Leader Unit | The First Hospital of China Medical University | | |
| Organizer | [D](../../../../D:/Users/Glacier/AppData/Local/Youdao/Dict/8.9.3.0/resultui/html/index.html#/javascript:;)epartment [of](../../../../D:/Users/Glacier/AppData/Local/Youdao/Dict/8.9.3.0/resultui/html/index.html#/javascript:;)  [a](../../../../D:/Users/Glacier/AppData/Local/Youdao/Dict/8.9.3.0/resultui/html/index.html#/javascript:;)nus [and](../../../../D:/Users/Glacier/AppData/Local/Youdao/Dict/8.9.3.0/resultui/html/index.html#/javascript:;) [intestine](../../../../D:/Users/Glacier/AppData/Local/Youdao/Dict/8.9.3.0/resultui/html/index.html#/javascript:;) [surgery](../../../../D:/Users/Glacier/AppData/Local/Youdao/Dict/8.9.3.0/resultui/html/index.html#/javascript:;) | Principal investigator | Chengzhong Xing |
| Categories of Review | Review | Mode | Quick Review |
| Conference information | | | |
| - Application Form of Ethical Review(Review) - Informed Consent Form | | | |
| 1.The Ethics Committee’s decision to review the project is as follows (put × in □)  ☒ Consent □Corrected consent □Corrected review □Dissent  2. After review by the Ethics Committee, informed consent is considered to be exempt.  3. Such ethical review and approval documents shall not be regarded as the basis for the registration of drugs and devices under the State Food and Drug Administration.  Director (Deputy director):  Official seal of Ethics Committee:  Data: | | | |
| During the implementation of the project, the applicant shall strictly abide by the following terms and conditions:   1. The ETHICS Committee decides that the follow-up review frequency of the project is 12 months. Please submit the annual follow-up review report one month in advance according to the review frequency. 2. If the principal investigator is changed during the study, a written explanation shall be submitted to the ETHICS Committee in time。 3. For any modification to the clinical study protocol, informed consent, recruitment materials, etc., apply for revision review of the study protocol, informed consent, etc. 4. When serious adverse events occur, timely submit a serious adverse event report; After an emergency report, please submit a detailed follow-up report of serious adverse events as soon as possible. 5. Immediately submit a written report to the Ethics Committee in case of any situation that may significantly affect the conduct of the study or increase the risk to the subject. 6. Failure to comply with protocol to conduct studies may adversely affect the rights/health of subjects and the scientific nature of the study, and a protocol violation review report shall be submitted. 7. Suspend or terminate the clinical study in advance, timely submit the project suspension, and terminate the study review report. 8. Complete the clinical study and submit the final review report. 9. This ethical review approval is valid for one year. If it is not carried out within the validity period, it shall be resubmitted to the Ethics Committee for review before the research is carried out. 10. Address of Ethics Committee: No. 155, Nanjing North Street, Heping District, Shenyang City, Liaoning Province, email: 110001, Office telephone: 023-83282837. | | | |

～～～～～～～～～～～～～～～～～END～～～～～～～～～～～～～～～～～
